# Using Functional Analysis as a Framework to Guide Individualized Treatment for Negative Symptoms

**DOI:** 10.3389/fpsyg.2017.02108

**Published:** 2017-12-05

**Authors:** Tania M. Lincoln, Marcel Riehle, Matthias Pillny, Sylvia Helbig-Lang, Anne-Katharina Fladung, Matthias Hartmann-Riemer, Stefan Kaiser

**Affiliations:** ^1^Clinical Psychology and Psychotherapy, Faculty of Psychology and Movement Sciences, Institute of Psychology, Universität Hamburg, Hamburg, Germany; ^2^Department of Psychiatry, Psychotherapy and Psychosomatics, Psychiatric Hospital, University of Zurich, Zurich, Switzerland; ^3^Adult Psychiatry Division, Department of Mental Health and Psychiatry, Geneva University Hospital, Geneva, Switzerland

**Keywords:** formulation, learning, consequences, reward, individualized intervention

## Abstract

Although numerous interventions are available for negative symptoms, outcomes have been unsatisfactory with pharmacological and psychological interventions producing changes of only limited clinical significance. Here, we argue that because negative symptoms occur as a complex syndrome caused and maintained by numerous factors that vary between individuals they are unlikely to be treated effectively by the present “one size fits all” approaches. Instead, a well-founded selection of those interventions relevant to each individual is needed to optimize both the efficiency and the efficacy of existing approaches. The concept of functional analysis (FA) can be used to structure existing knowledge so that it can guide individualized treatment planning. FA is based on stimulus—response learning mechanisms taking into account the characteristics of the organism that contribute to the responses, their consequences and the contingency with which consequences are tied to the response. FA can thus be flexibly applied to the level of individual patients to understand the factors causing and maintaining negative symptoms and derive suitable interventions. In this article we will briefly introduce the concept of FA and demonstrate—exemplarily—how known psychological and biological correlates of negative symptoms can be incorporated into its framework. We then outline the framework's implications for individual assessment and treatment. Following the logic of FA, we argue that a detailed assessment is needed to identify the key factors causing or maintaining negative symptoms for each individual patient. Interventions can then be selected according to their likelihood of changing these key factors and need to take interactions between different factors into account. Supplementary case vignettes exemplify the usefulness of functional analysis for individual treatment planning. Finally, we discuss and point to avenues for future research guided by this model.

## Introduction

Negative symptoms of schizophrenia are characterized by blunted affect, alogia, asociality, anhedonia, and apathy/avolition (Kirkpatrick et al., 2006). With a prevalence of about 60% negative symptoms are as frequent as hallucinations in patients with schizophrenia spectrum disorders (Bobes et al., 2010) and are associated with marked impairments in social functioning and quality of life (Fervaha et al., 2014). Recent conceptualizations see negative symptoms as a two-dimensional construct, comprised of symptoms related to diminished motivation and symptoms related to diminished expression (Horan et al., 2011; Strauss et al., 2012). Diminished motivation describes a lack of motivation to engage in or sustain goal-directed behavior (apathy, avolition, asociality, anhedonia). Diminished expression involves reduced expression in several domains of non-verbal and verbal communication, such as facial expression, vocal prosody, gesturing, and quantity of spoken words.

Although negative symptoms have received considerably less attention than positive symptoms, they are recognized to be a distinct and important therapeutic domain (Kirkpatrick et al., 2006). Disappointingly though, they have proven notoriously difficult to treat. Recent reviews of the effectiveness of interventions aimed at negative symptoms report some positive effects of cognitive therapy and various skill trainings but the findings are mixed and the small effects can rarely be replicated in samples of patients with more pronounced negative symptoms (Aleman et al., 2017; Riehle et al., 2017). Similarly, despite a couple of promising studies, biological approaches, such as medication and electromagnetic neurostimulation have not been successful in reducing negative symptoms and there is even some indication that antipsychotics can potentially aggravate negative symptoms (Aleman et al., 2017). This state of affairs has led to an overwhelming consensus in the field that increased effort is required to enhance the treatment of negative symptoms.

We argue that the lack of effectiveness of existing approaches is likely to be due to the fact that we are dealing with a complex syndrome caused and maintained by numerous factors that vary between individuals. Because of their complexity on the phenomenological and etiological level negative symptoms are unlikely to be “solved” by focusing on single aspects alone or by a “one size fits all” approach. At present, however, most interventions target single deficits related to negative symptoms and are offered to patients without assessing whether the deficit at focus is causing or maintaining the individual patient's symptoms. For example, a frequent target has been neurocognitive impairment that is known to be associated with negative symptoms (Ventura et al., 2009; Strauss et al., 2012). However, despite producing improvements on neurocognition, cognitive remediation on its own has small effects—at the most—on negative symptoms (Elis et al., 2013; Cella et al., 2017; Riehle et al., 2017). Interestingly, however, its effects appear to be somewhat stronger if it is administered along with additional components that address social skills or problem solving (Roder et al., 2011; Eak et al., 2013; Mueller et al., 2017). Similarly, social skills as a stand-alone have produced small and mixed effects for negative symptoms, but seem to impact on negative symptoms if combined with other skill-trainings and cognitive therapy (Granholm et al., 2013, 2014). Integrated packages may show stronger effects because broader approaches include modules relevant to a larger group of patients or because they take interactions between different factors relevant to negative symptoms into account.

However, as offering all interventions available to every patient is neither feasible nor efficient, we argue that a well-founded selection of those interventions relevant to each individual is likely to optimize both the efficiency and the efficacy of existing approaches. Another interesting observation is that the effectiveness of existing approaches appears to differ depending on the dimension of negative symptoms. In a recent review of randomized controlled trials of psychological approaches targeting negative symptoms (Riehle et al., 2017) the authors found that the effect on the motivational components of negative symptoms was largest in studies employing cognitive behavior therapy and/or social skills trainings whereas the effect on the expressive components of negative symptoms was largest in studies using non-verbal interventions such as body-oriented psychotherapy. This indicates that selection of interventions also should be guided by a well-founded understanding of which aspect of negative symptoms needs to be addressed in order to receive the best outcomes in terms of functioning and recovery. Finally, the importance of manipulating the stimulus and context structure in cognitive rehabilitation has been emphasized (Silverstein et al., 2012) but not put into practice so far.

Here, we argue that the concept of functional analysis (FA) can be used to structure existing knowledge so that it can be used to move beyond the “one size fits all” approaches and guide individualized treatment planning. FA can be flexibly applied to the level of individual patients to understand the factors causing and maintaining the symptoms. In this article we will briefly introduce the concept of FA and demonstrate—exemplarily—how psychological and biological correlates of negative symptoms can be incorporated into its framework. We then outline the framework's implications for individual assessment and treatment.

## General introduction to functional analysis

FA has its origins in early behavior therapy and is used to identify important variables that cause and maintain behavior. The work by Watson that focused on stimulus (S)-response (R) mechanisms and particularly Skinner's S-R-Consequence (C) models of behavior, inspired the development of different behavior analysis procedures and their application to mental health problems. One line of research is best described as “experimental functional analysis” and is associated with multiple researchers, such as Iwata, Lovaas, Carr, Bijou (for a summary of this tradition see Dixon et al., 2012). These researchers strongly relied on the observation of individual behavior after experimentally manipulating environmental variables related to this behavior. In a series of experimental case studies, researchers in this tradition of FA demonstrated that maladaptive behavior can be explained as functional responses to environmental stimuli, in particular by the consequences following behavior (e.g., Lovaas et al., 1965; Carr et al., 1976). Seemingly independently of this group of researchers, Kanfer and Saslow (1965) developed a model of functional analysis that, while also based on S-R-C learning mechanisms, takes into account a person's characteristics that contribute to the responses. This conceptualization thus incorporates the pre-existing differences between individuals in biological disposition and any other differences resulting from learning history and thereby provides a framework to (theoretically) fully explain a behavioral response in any given situation. Although this conceptualization is widely used for the assessment and treatment planning of mental health problems and has strongly influenced cognitive behavioral models and interventions for several mental disorders (particularly anxiety disorders), so far it has not been applied to negative symptoms, which is the focus of the present article.

In the following we lean on the SORCK-conceptualization that Kanfer and colleagues began to develop in 1965 and have since elaborated further (Kanfer et al., 2012). The questions that this model aims to answer are: Under which circumstances did the behavior develop and which factors are triggering and maintaining it at the moment? The first question addresses a macro-analytic perspective on factors contributing to the onset of a problematic behavior, while the second focuses on the micro-level of specific situations in which the problematic behavior occurs and is maintained.

**Figure 1:**
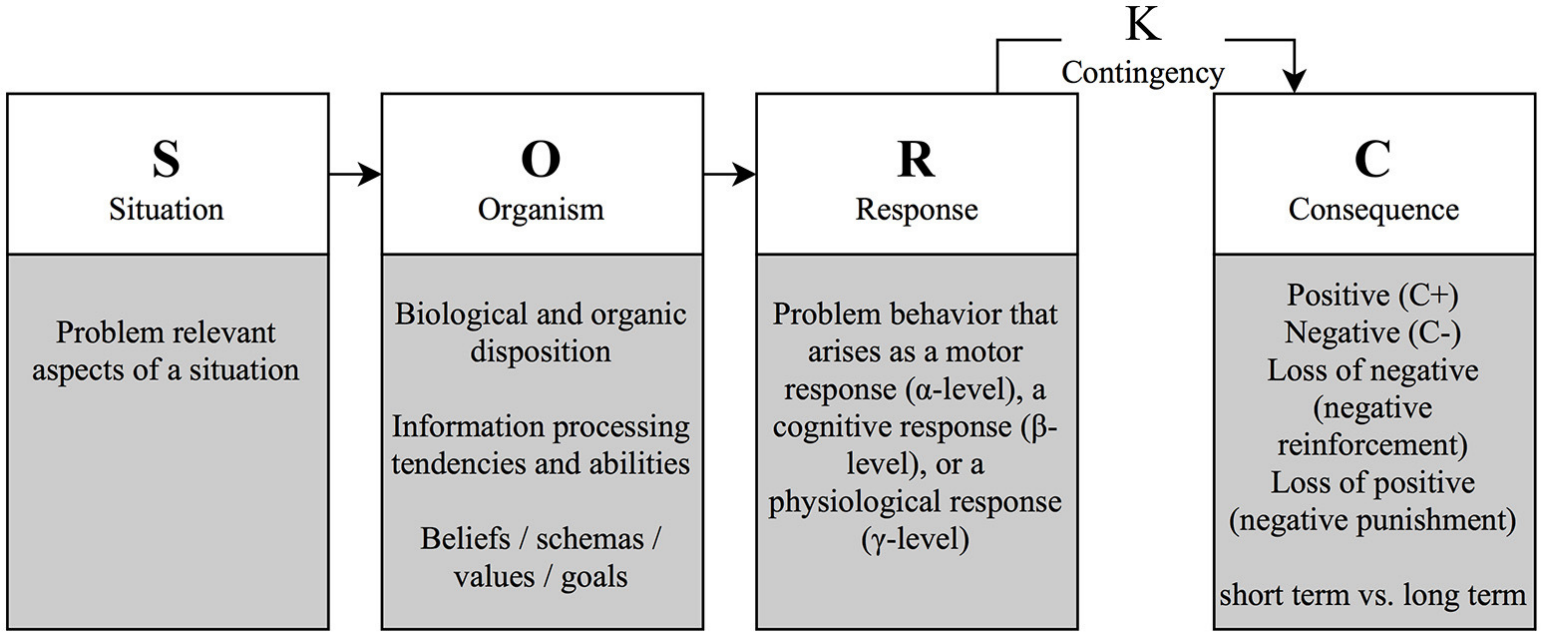
Functional analysis model.

As depicted in Figure 1, the model explains a problematic response at the micro-level of *specific situations (S)*, which are conceptualized as antecedents of the problematic response. Situational factors include problem relevant aspects of the situation, in which the problem arises, such as time, place, or presence of relevant others.

*Problematic responses (R)* can be categorized into behavior excesses, deficits or inappropriate behavior and are described at the level of visible behavior (motor-behavioral α-level), automatic thoughts (cognitive β-level), and at the psychophysiological level (γ-level) (Lang, 1968). Thus, the problem response can be understood as a conglomerate of different manifestations of a reaction that can, but do not necessarily, correspond with each other. Although the authors stress the relevance of emotions in the formation and maintenance of behavior, they do not specify an emotional level, arguing that emotions result from the physiological, motor, and cognitive responses (Kanfer et al., 2012).

The *organism variable (O)* mediates between the situational stimuli (S) and the response (R) and includes the learning history (β-variables), somatic and biological conditions (γ-variables) and their interaction. Thus, the O-variable can include physical conditions, such as being hungry, tired, or ill, dispositions, such as intelligence or personality traits, information processing tendencies, such as cognitive biases, but also individual standards, general beliefs, expectations, and goals that result from a person's learning history.

The *consequences (C)* regulate behavior by means of reinforcement or punishment (Skinner, 1953). Four qualities of consequences are commonly distinguished: Positive and negative consequences that emerge are generally described as reinforcers and punishers, respectively. Furthermore, both negative and positive consequences can be canceled or cease to exist, which is termed negative reinforcement and negative punishment (also called “punishment by contingent withdrawal”), respectively. Both types of reinforcers increase the likelihood of showing a behavior again in the future, whereas both types of punishers decrease this likelihood. Consequences might be internal (e.g., reduction in aversive feelings) or external (e.g., changes in environmental factors or behavior of others).

The *contingencies (K)* describe the timing and the likelihood of a specific consequence that follows a particular behavior. Consequences can be immediate vs. delayed, regular, or sporadic and the type of association can be non-discriminative or discriminative (if it only occurs when specific stimuli are present in a situation). Consequences that occur regularly after a behavior improve the learning of a new behavior, while sporadically occurring consequences are more likely to maintain the response. Additionally, immediate consequences generally are more relevant to learning than delayed ones. Beyond the contingencies, the strength of the effect of the consequences on future responses is also determined by how meaningful the consequence is for the person involved, which is determined by a person's goals, values, preferences, and satiation or deprivation.

## Feedback loops

**Figure 2:**
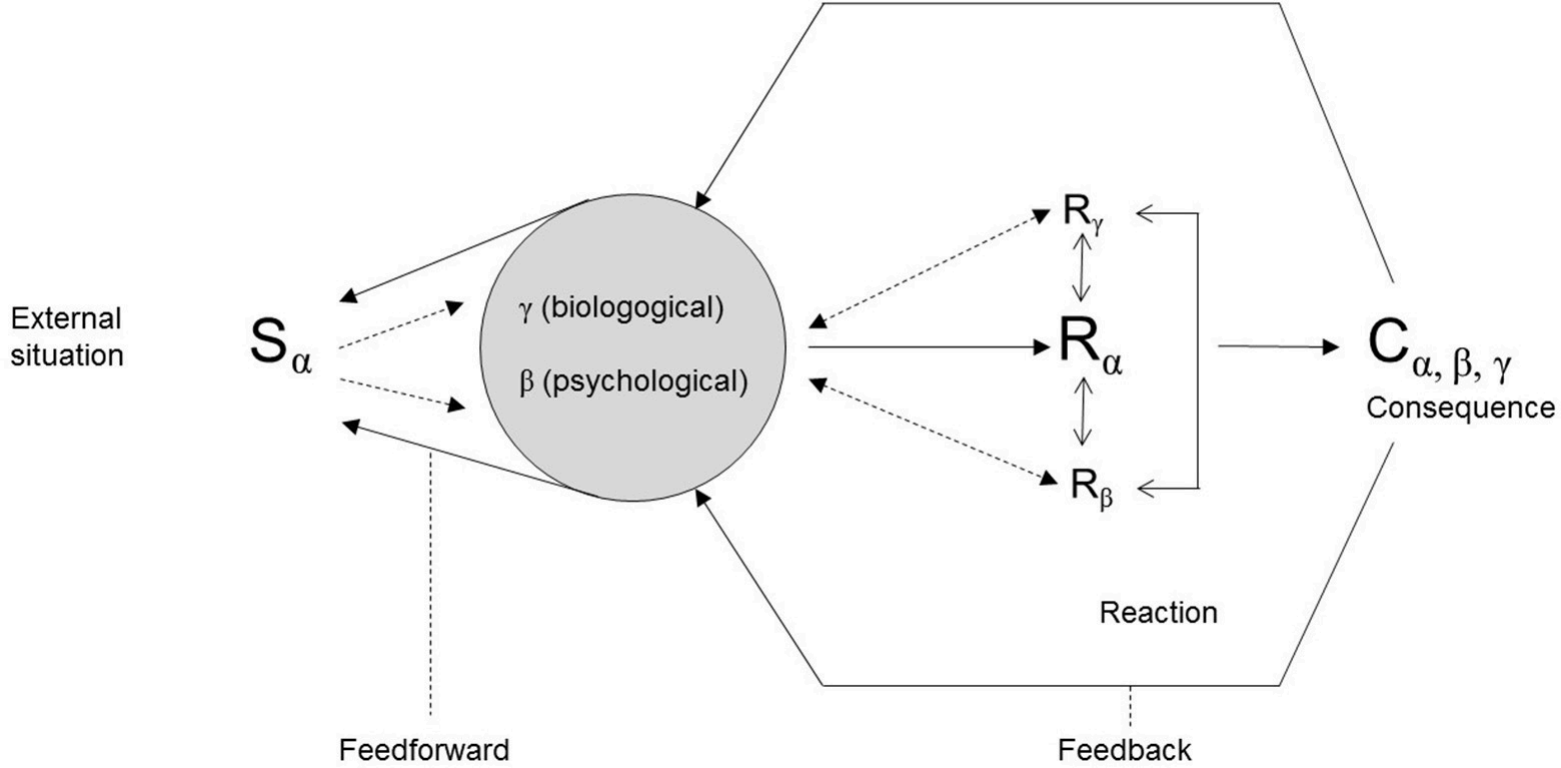
A self-regulation model of behavior (mod. after Kanfer and Karoly, 1972).

While the first generation of FA models assumed a linear progression of S, R, and C, the subsequent developments were complemented by a stronger focus on interactions between the variables and further specification of each of the variables. An example of such a model is the self-regulation model by Kanfer and Karoly (1972) (see Figure 2).

This model includes feedback-/feedforward-processes between the O and the S, the R and the O, and the C and the O variable. For example, the feedforward-loop from O to S, indicates that previous experiences or cognitive biases can influence the way a situation is perceived and thus change the situation. The feedback loops also take into account that consequences exert their influence by feeding back into the organism variable and thus also into future situational variables. More complex behaviors can then be described as a reaction chain in which consequences from one problematic behavior constitute situational triggers for another problematic behavior.

## Incorporating negative symptoms into the functional analysis framework

Although the FA is generally used to explain specific behavior of a specific individual, we will begin by using it as a framework to explain negative symptoms on a more general level (including macro- and micro-level perspectives). We will briefly specify what constitutes the problem behavior in negative symptoms in the R variable. We will then provide examples of research findings relevant to the S and O-variable in negative symptoms, before elaborating on consequences and contingency issues. Feedback mechanisms will be specified where appropriate. A summary of the exemplary factors considered potentially relevant to each level of explanation is provided in Table 1. Note that our literature selection was not the result of a systematic review. We based the selection of which findings to incorporate on a thorough knowledge of existing literature with a focus on systematic reviews and included a heterogeneous group of authors to secure expert knowledge form different domains. However, the state of research presented for each of the variables is open to further suggestions, extensions, or different accentuations. At which level of the FA best to categorize some of the existing research findings also required discussion so that the proposed solution is based on a consensus.

**Table 1:** Example of how existing knowledge on negative symptoms can be incorporated into the framework of a Functional Analysis.

**S situation**	**O organism**	**R response**	**C consequences**
**Micro-level:** Specific situational trigger (e.g., ringing alarm clock, being approached by another person, being alone at home, etc.) **Macro-level:** Understimulating or overstimulating and distressing environment (e.g., institutional deprivation, trauma, social adversity, low socio-economic status, discrimination, poor social network, low social support, social undermining, criticism and stigmatization)	β**-level Neurocognitive deficits:** e.g., impaired processing speed, attention/vigilance, working memory, verbal memory, reasoning and problem solving, verbal comprehension **Social cognition:** e.g., impairment in Theory of Mind, impairments in identification and discrimination of facial-emotional expressions, impaired awareness of social inference, lower competence in relationship perception and conveying of empathy **Enduring schemas about self and others:** e.g., low self-esteem, reduced sense of self-efficacy, beliefs of asociality, low expectancies of success, and pleasure, attachment insecurity **Anticipation of pleasure and reward:** e.g., reduced anticipation of positive affect and positive outcomes (rewards) **Reward representation and decision-making:** e.g., degraded representation of value, overestimation of the costs of effort to attain reward, difficulties in generating options for action **Reinforcement learning:** Impaired learning from positive consequences γ**-level** e.g., reduced physical fitness, increased prevalence for medical illnesses, immune functioning, side-effects of antipsychotic medication, substance abuse, genetic predispositions	α-**level** **Diminished motivation:** Lack of initiation or persistence in goal-directed daily activities, such as going to work/school, self-care, pursuing hobbies, or interactions with friends and family **Diminished expression:** e.g., reduced verbal behavior, altered vocal prosody, reduced non-verbal communication (e.g., reduced positive facial expression such as smiling), reduced gesturing (e.g., head nodding or forward leaning), impaired synchronization of interpersonal movements expressive behavior β-**level** e.g., negative automatic thoughts (e.g., “I don't have what it takes to do this”, “I won't have fun”, “It won't work out for me anyway”, “It's not worth trying”) γ-**level** e.g., reduced muscle activity (e.g., smiling muscles), increased autonomic and HPA axis responses	**Positive reinforcement:** Increased effort from interaction partners, increased attention from mental-health professionals, social support, practical and financial help (e.g., disability pensions) **Negative reinforcement:** Reduction of fear, worry, or tension, short-term relief from social and affective consequences **Punishment:** Social rejection, criticism, Health-related problems **Negative punishment:** Loss of social achievement, Less positive affective responses and liking from interaction partners, Loss of social interaction, Fewer pleasurable events, Less experience of success, Non-achievement of approach goals

## The R-variable—prototypical problem behavior

As implied in the adjective “negative,” negative symptoms are characterized by an *absence* of observable behavior (α-level). The motivational subdomain manifests as a lack of initiation of or persistence in goal-directed activities (Trémeau et al., 2012; Docx et al., 2013; Walther et al., 2015), such as getting up to go to work/school, getting dressed, or combing one's hair, going to see a football game, or talking to the neighbor self-care. The expressiveness subdomain manifests as a reduction of verbal behavior (e.g., one syllable answers) and of non-verbal behavior, such as facial expressions, vocal prosody, and gesturing. What can be observed is a lack of smiling, head nodding, or forward leaning (Lavelle et al., 2014) and impaired synchronization of interpersonal movements and facial mimicry (Kupper et al., 2015; Riehle and Lincoln, 2016). An example for the cognitive response (β-level) are dysfunctional beliefs that patients with negative symptoms tend to report, such as low expectations of success and pleasure (Gard et al., 2007), and defeatist performance beliefs (Rector, 2004) that are likely to be reflected in concrete automatic negative thoughts in a given situation, (e.g., “I don't have what it takes to do this,” “I won't have fun,” “It's not worth trying”) (Grant and Beck, 2009; Horan et al., 2010). An example of the physiological response (γ-level) could be an increased heart rate as indicated by research showing negative symptoms to be associated with increased heart rate reactivity to stressors (Brekke et al., 1995). However, more research on physiological responses (in contrast to resting-states) is needed in samples with negative symptoms.

## The S-variable—relevant situational conditions

At the micro-level of an individual FA the S-variable can be practically any situation a person encounters, such as responding to a signal from the alarm clock indicating that it is time to get up. If we move to the macro-level that explains individual behavior in the context of enduring external influences, there is indication that prolonged exposure to either understimulation, in terms of a depriving environment, or to overstimulation, in terms of social adversity and stress, is linked to negative symptoms. For example, patients who were institutionalized over long periods of time were found to show decreased spontaneity, reduced curiosity, reduced drive to interact, and blunted affect (Barton, 1959; Wing and Brown, 1970; Oshima et al., 2005). Moreover, discharge from long-term inpatient forensic facilities was followed by an improvement of negative symptoms (Lincoln et al., 2005). Other research shows that social adversity, including social undermining, low levels of social support, smaller networks, trauma and discrimination, and low-socio-economic status are associated with higher levels of negative symptoms (Lysaker et al., 2006; Jaya et al., 2017).

According to the feedback model outlined in Figure 2, situational factors are characterized by their close interaction with the O-variable. In this regard it can be speculated that persistent situational factors such as social deprivation will have a detrimental effect on a person's cognitive abilities. For example, cognitive functioning was shown to improve in people with serious mental illness if they were removed from homeless shelters to group homes or independent apartments (Seidman et al., 2003). Moreover, ongoing social adversity tends to influence a person's beliefs related to the self and others (Jaya et al., 2017), attachment styles (Sitko et al., 2014) and ability to regulate emotions (Lincoln et al., 2017). Situational feedforward loops from O to S can arise from *y*-level variables, for example if a physical condition, such as obesity, reduces the likelihood of being approached by someone from the other sex or from β-level variables in the sense that impaired attention and working memory can impact on the way a situation is processed.

## The O-variable—relevant person-level factors

As can be seen in Table 1, most of what we know from research on negative symptoms can be conceptualized at the β-level (cognition) of the O-variable. This includes *neurocognitive deficits* which have been linked to reduced expressiveness in particular (Cohen et al., 2012, 2013; Chang et al., 2014; Hartmann-Riemer et al., 2015) suggesting that the cognitive resources required to express emotions via gestures, facial expressions, and vocal features are limited in people with schizophrenia (Cohen et al., 2012, 2014). Another β-level domain is *social cognition* that describes the mental operations crucial for social interactions (Green et al., 2015a). For example, impairments in Theory of Mind (or mentalizing) are clearly associated with negative symptoms (Brüne, 2005), as are difficulties in identifying and discriminating facial expressions of emotions (Kohler et al., 2010; Savla et al., 2013). Moreover, *enduring schemas about self*, such as low self-esteem (Lincoln et al., 2011; Palmier-Claus et al., 2011), a reduced sense of self-efficacy (Bentall et al., 2010), and attachment insecurity (Gumley et al., 2014) associated with negative symptoms can be subsumed at the β-level of the O-variable. Other examples of β-level variables are the *ability to anticipate pleasure and reward* (Juckel et al., 2006; Gard et al., 2007; Engel et al., 2016), *deficits in generating, maintaining, and updating mental representations of value, effort-based decision making* (Gold et al., 2013; Barch et al., 2014; Green et al., 2015b; Hartmann et al., 2015) and *difficulties in reinforcement learning* from positive outcomes (Strauss et al., 2011; Gold et al., 2012), which all add to explaining the reduction in goal-directed behavior observable at the R-level. Although these variables are presented separately in Table 1, note that they are closely related and influence each other. For example, anticipation deficits related to positive affect might reflect a deficit in anticipating rewards or result from the dysfunctional beliefs described above. Moreover, reinforcement learning deficits might at least partially be driven by impaired working memory capacity (Collins et al., 2014).

Aspects relevant to the γ-level (physiology) of the O-variable include physical fitness, chronic physical illnesses, functioning of the autonomic, HPA and immune system as well as effects of medication (e.g., sedation, drowsiness) and other substances. Moreover, genetic and epigenetic factors can be subsumed here. To provide an example, reduced levels of testosterone and increased levels of cortisol, ACTH and electrodermal responses are found to be associated with negative symptoms (Maina et al., 1995; Shirayama et al., 2002; Zhang et al., 2005). Moreover, patients with negative symptoms tend to show reduced cardiorespiratory fitness even in the absence of physical illness (Vancampfort et al., 2015) and higher rates of metabolic syndrome (Sicras-Mainar et al., 2015). In terms of interactions, physical health problems and negative symptoms might bi-directionally influence each other (Sicras-Mainar et al., 2015), in the sense that physical impairment will lead to lower activity levels on the α-level of the R-variable and that repeated inactivity on the α-level of the R-variable can contribute to a sedentary lifestyle, which in turn increases the risk of cardiovascular disease. As a second example, medication effects on somnolence, drowsiness, and fatigue can have considerable impact on behavioral and cognitive responses (Carpenter et al., 1985). Moreover, medication induced akinesia or bradykinesia can affect expressive behaviors. Finally, antipsychotics have been suggested to directly cause amotivation and subsequent impairments in goal-directed behavior by the reduction of dopaminergic transmission (Ikemoto et al., 2015).

## The C-variable—consequences

Recall that in FA, only reinforcing consequences are linked to the maintenance of problematic behavior, since punishing consequences will decrease the likelihood of the occurrence of the problem behavior. Although problem behavior will also be followed by negative consequences, particularly in the long run, from a therapeutic perspective it seems most fruitful to gain a full understanding of the reinforcing factors that could be contributing to its maintenance. In terms of positive reinforcers the social withdrawal and inactivity that are part of the negative symptom response are likely to increase efforts in interaction partners and, particularly, mental-health professionals to provide support and help. This can occur in terms of increased attention and care, particularly early on in the course of the disorder when social ties are still more preserved. Social support, practical help, and financial support (e.g., in form of disability pensions) may also be subsumed here. Nevertheless, given that anticipation of pleasure and learning from rewards is impaired in negative symptoms, we argue that negative rather than positive reinforcement is likely to be the predominant mechanism to maintain negative symptoms. Both withdrawing from social interactions and withdrawing from other kinds of effortful activity are likely to be reinforced by a reduction of fear, worry, or tension, which may be experienced as a short-term relief from the perceived or anticipated burden of effort or responsibility for one's actions along with its risk of failure. This mechanism can be expected to be much stronger in patients with negative symptoms who are more likely to expect failure (see O-variable section on beliefs and anticipation). The mechanism of negative reinforcement of negative symptoms could hardly be expressed more clearly than in a description by a former patient with negative symptoms, who stated that “giving up, refusing to hope, not trying, not caring: all of these were ways to try and protect the last fragile traces of our spirit and our selfhood from undergoing another crushing” (Deegan, 2016). Accordingly, some authors have suggested that negative symptoms play an adaptive role by protecting patients from further stressors and trauma (Brekke et al., 1995; Beck et al., 2009; White et al., 2013; Velligan et al., 2014) and by protecting against suicidal ideation and attempts (Fenton et al., 1997; Hawton et al., 2005).

Another consequence of negative symptom problem behavior is the loss of positive reinforcement in various domains, including social, achievement, and pleasure related domains (Gard et al., 2007, 2014). This includes rejection and criticism by interaction partners (Domínguez-Martínez et al., 2014) who tend to gradually withdraw (Lavelle et al., 2013; Riehle et al., 2015; Riehle and Lincoln, 2016) leaving patients with reduced opportunities to improve in social cognition and social skills, or achievement related successes (e.g., win at a board game, receive job offers, receive promotions, monetary rewards etc.), which is likely to nourish self-schemas and deficits in social and cognitive domains at the level of the O-variable (feedback loop). Finally, because the problem behavior is an absence of behavior rather than an excess (e.g., *not* initiating a social contact, *not* smiling etc.), it is essential to also look at the consequences of showing the functional behavior (e.g., initiating a social contact, smiling etc.). Possibly, functional behavior is not being as strongly reinforced as it is in healthy individuals.

## The K-variable—contingencies

Interestingly, the overall picture in regard to consequences is that the problem behavior is persistent despite the numerous negative consequences and the dearth of positive ones. This becomes more understandable by examining the contingencies that describe the timing and the likelihood of the consequences. Since the reinforcing consequences (e.g., reduction of negative affect) tend to follow the problem behavior immediately and regularly, they are likely to exert a stronger influence over it than the punishing ones (e.g., rejection, health-related problems) that tend to follow with a long delay and a certain irregularity. Another contingency-related issue is that the strength of the effect of the consequences on future responses is determined by how meaningful the consequence is for the person involved, which is determined by a person's goals, values, and preferences. For example, as negative symptoms are associated with having relatively more avoidance than approach related goals (Schlier et al., 2017) these patients might value reinforcers of approach-goal directed behavior less and thus learn less from them.

## Implications for assessment

As outlined above, the FA serves as a basis to derive interventions that target the factors that cause and maintain negative symptoms in a given individual. To arrive at optimal effectiveness, treatment planning should take place for every individual after a thorough assessment of potential problems in each of the variables outlined above. An overview is presented in Table 2. Over and above validated assessment tools for negative symptoms (for an overview of existing assessment tools for negative symptoms see Lincoln et al., 2016) it is advisable to assess prototypical situations in collaboration with the patient (as exemplified in the case vignettes), as these provide information on concrete behavioral responses (R) and can also be used to identify the situational factors (S) and the specific consequences (C). If available, ambulatory assessment devices (Myin-Germeys et al., 2011) are likely to provide valuable information on situational triggers of problem vs. functional behavior along with the consequences and contingencies. In regard to understanding past and ongoing situational factors with relevance to the O-variable, knowledge of childhood and adolescent adversity (e.g., life-history charts) and the present living and working situation is relevant. As an assessment of all variables on the level of the O-variable goes beyond the scope of any clinician, a practical stance needs to be adopted here, focusing on those aspects deemed most relevant for a given patient: neurocognitive deficits may be assessed by a standardized neuropsychological test-battery, such as the MATRICS, that also tests for some social-cognitive skills and emotion processing (Nuechterlein et al., 2008). Social-interactional and affiliate skills are best assessed by standardized interactional probes (e.g., Blanchard et al., 2015); a scale to assess a range of dysfunctional beliefs specific to negative symptoms is currently under development (Pillny and Lincoln, under review). Regarding reward processing there are some questionnaires that can be used to assess anticipatory pleasure but standardized laboratory tests or other measures that allow to identify individual dysfunctions of reward anticipation, value representation, or effort discounting for the purpose of treatment planning are yet to be developed. For the γ-level (physiology) of the O-variable, a medical assessment should involve a thorough physical history and examination and elucidate the effects of antipsychotic medication and substance use on negative symptoms. Based on this assessment, the therapist uses the FA framework and develops a preliminary working model of the most relevant factors and their interactions that are driving and maintaining the problem behavior.

**Table 2:** Overview of relevant treatment targets, methods of assessment, therapeutic aims, and potential as well as existing interventions.

**Treatment target**	**Assessment**	**Therapeutic aims**	**Evidence based and potentially helpful interventions**
**SITUATION (S)**			
Prototypical triggers; Persistent unfavorable conditions (e.g., social adversity, poor social support, etc.)	Ambulatory assessment devices, open interviews, Questionnaires, life-history charts, informant information	Reduction of adverse situations (e.g., understimulation), reduction of social and environmental stressors	Family interventions (e.g., communication training, crisis management, psycho-education) that aim to reduce psychosocial stress Providing environmental supports to prompt initiation and persistence of goal directed behavior. Examples for this are provided in one module of the Motivation and Enhancement Training for Negative Symptoms (MOVE; Velligan et al., 2015) Avenues for further research: anti-stigmatization, encouraging, and providing opportunities for employment and networking
**ORGANISM (O)**			
***ß*****-level** Neurocognitive deficits	Comprehensive neuropsychological test battery (e.g., MATRICS, Nuechterlein et al., 2008) or interview-based assessment	Improvement of attention, memory, executive functions	Cognitive skill trainings (or: cognitive remediation), if possible combined with motivational components or broader skill trainings. Examples can be found in the Cognitive Enhancement Training (Eak et al., 2013) or the Integrated Psychological Therapy (Roder et al., 2006). Training of affect recognition (e.g., Wölwer et al., 2005); Social Cognition and Interaction Training (Roberts et al., 2014); Training of Emotion Processing (e.g., Bechi et al., 2012); Training to improve on Theory of Mind (e.g., Mazza et al., 2010)
Social cognition and social interaction	Diagnostic role-plays, behavioral tests, self-report questionnaires, informant information	Improvement of affect recognition, processing of social information (e.g., theory of mind, empathy)	
Dysfunctional beliefs, negative self-concepts, and attachment insecurity	Self-report scales, interview-based scales	Changing negative schemas about self and others	Avenues for further research: schema therapy, attachment focused interventions, cognitive interventions (e.g. Grant et al., 2012)
Reward processing	Self-report scales to assess anticipatory pleasure, Laboratory tests to assess processes (e.g., Pizzagalli et al., 2005; Hartmann et al., 2015)	Anticipation of positive emotions, improving value representation and learning from positive reinforcement	Training anticipatory pleasure skills. Evidence-based examples are the training by Favrod et al. (2010); or the module on anticipatory pleasure from the MOVE-program (Velligan et al., 2015); Interventions that increase awareness of present positive states, such as Acceptance and Commitment Therapy (White et al., 2011) and loving-kindness meditation (Johnson et al., 2011) Avenues for further research: behavioral experiments using ambulatory interventions; augmentation of dopamine neurotransmission with stimulant drugs; pharmacological treatment with an antidepressant.
**y-level** Adverse medical conditions	Medical assessment	Improvement of physical health	Treatment of medical diseases, exercise, decrease of substance abuse, nutrition programs, yoga
**RESPONSE (R)**			
Negative symptoms	Self- and observated scales; diagnostic role-plays Informant information, observer-rated scales	Increase affiliate signals, correct dysfunctional automatic thoughts, increase active behavior	Behavioral activation (e.g., Jacobson et al., 2001); Social skill training (e.g., Bellack et al., 2004), trainings of affective display and non-verbal communication, such as mirror exercises (e.g. Schwartz et al., 2006; Velligan et al., 2014); cognitive interventions that challenge automatic thoughts (e.g. Grant et al., 2012) Avenues for further research: bio- and other automated movement feedback of facial expressions
**CONSEQUENCES (C)**			
Maintenance of problem behavior	Interview based assessment, ambulatory assessment, behavioral observation	Increase goal-directed, functional behavior	Avenues for research: individual schedules of reinforcement that reduce reinforcement of dysfunctional and increase reinforcement of functional behavior that can be incorporated into ward-based or family interventions (contingency management)

## Implications for therapy

In a next step, this model is discussed with the patient and suitable interventions can be collaboratively delineated. Following the logic of the FA, priority should be given to key causing or maintaining factors and interventions, or combinations of interventions, that are likely to change these factors. We will begin by reflecting on which type of intervention may be helpful to address each of the different FA components. Where available, we will point to existing interventions and note their evidence-base, but we will also make suggestions for novel and potentially suitable interventions. Again, however, we do not claim to have listed all of the possible interventions, although—to our knowledge—we mention all of the existing psychological approaches for negative symptoms. Moreover, we will address how interactions between different levels may be taken into account. Table 2 provides an overview of therapy-relevant targets according to their focus within the FA conceptualization, therapeutic aims, and (potentially) effective interventions. Three case examples in the supplement exemplify how the approach can be applied to individual patients.

## Interventions at the situational level (S)

The basic aim at this level is to reduce situations that trigger problem responses, such as environmental under-stimulation or social adversity and increase those that are likely to trigger a healthier response. This could mean supporting a move out of the institution and increasing social encounters for one patient or decreasing social stressors for another. If the patient is living with his or her family, psychoeducation, or skill-based family interventions that aim at reducing the stressful emotional climate in the family (Dixon et al., 2000) could be considered at this level as these types of interventions have been demonstrated effective in improving negative symptoms (Elis et al., 2013).

## Interventions at the level of the organism variable (O)

Depending on the individual case, the aim of interventions at the ß-level of the O-variable could be to improve on social-cognitive or neurocognitive skills and dysfunctional beliefs, to correct dysfunctional self- and interpersonal concepts, or to improve emotion- and reward processing as well as learning.

Neurocognition has long been targeted using cognitive remediation that typically includes training of attention, memory, and executive functions and has been shown effective for improving neurocognition (Elis et al., 2013). Trainings of affect recognition (e.g., Wölwer et al., 2005) and Theory of Mind (e.g., Mazza et al., 2010; Bechi et al., 2012; Roberts et al., 2014) are suitable to target problems in the domain of social cognition (Green et al., 2015a; Bonfils et al., 2016). These types of interventions are also included in the integrative concepts of Motivation and Enhancement Training (MOVE) (Velligan et al., 2015) and the Emotion and Imitation Training (Mazza et al., 2010), but rather than running each patient through the entire program, the FA approach would suggest to select the interventions according to the individual formulation. To address anticipatory pleasure, behavioral experiments may be helpful as a patient's expectation of achievable pleasure prior to an activity can be compared to the actual degree of achieved pleasure during the activity, such as done in the MOVE program (e.g., Velligan et al., 2015). Moreover, Favrod et al. (2010, 2015) have developed an anticipatory pleasure skills training that uses a positive imagination technique to generate pleasure from anticipating activities and behavioral activation homework exercises to get people to engage in the respective activities. A novel approach would be to combine ambulatory assessment (i.e., monitoring levels of effort and pleasure in regard to momentary and upcoming events in a daily life) and ambulatory intervention (i.e., comparing anticipated effort and pleasure with the pleasure actually experienced, scheduling activity plans based on previous experiences and being sent reminder prompts and reinforcers to encourage and reinforce goal directed behavior). Another potentially helpful intervention is to increase awareness of momentary emotional states. In support of this, mindfulness based interventions, acceptance and commitment therapy and loving-kindness-meditation show promising effects for negative symptoms via increasing awareness of affective states (White et al., 2011) and positive affect (Johnson et al., 2011).

Since there is increasing knowledge about the neurobiological basis of the reward system and its dysfunction in schizophrenia, biological approaches to improve anticipation of reward/pleasure seem promising in theory and a number of potential avenues should be mentioned. First, an augmentation of dopamine neurotransmission with stimulant drugs has been shown to modulate effort-based decision-making and striatal responses in healthy volunteers (Wardle et al., 2011), but so far, there is no clear evidence for the treatment of negative symptoms. Second, in patients with depressive disorder pharmacological treatment with an antidepressant has been shown to enhance striatal responses to reward anticipation along with severity of depression (Stoy et al., 2012), thus this may be an effective approach for people with negative symptoms as well. Finally, a promising approach to improve on reward processing is to use non-invasive brain stimulation with transcranial magnetic stimulation (TMS) or transcranial direct current stimulation (tDCS) of the prefrontal cortex to remotely stimulate the interconnected striatal and ventral midbrain areas (Strafella et al., 2001; Chib et al., 2013). This modulation may improve reward anticipation or reinforcement learning and is currently being investigated with respect to negative symptoms (Chib, 2014).

The only type of intervention so far to directly target the dysfunctional beliefs in patients with negative symptoms is cognitive behavioral therapy for psychosis (CBTp). One randomized controlled trial of CBTp (Grant et al., 2012) focused primarily on changing generalized beliefs that hinder goal-attainment in patients with prominent negative symptoms and found improvements for apathy, avolition, and functioning but not for anhedonia, flat affect, and alogia. Relevantly, a subsequent small uncontrolled pilot trial (Staring et al., 2013) using the same approach found the pre- to post effect for negative symptoms to be partially mediated by a change in dysfunctional beliefs. This underscores the relevance of dysfunctional beliefs on the O-levels to negative symptoms at the R-level. However, following the logic of the FA model, these interventions are even more likely to be effective if patients are preselected for dysfunctional beliefs (as indicated in work by Granholm et al., 2013) and if they are combined with interventions at the other levels of the model. So far, to our knowledge, there are no interventions that primarily focus on enduring schemas about self- and others, such as early maladaptive schemas or attachment in the context of negative symptoms. Approaches that target these issues in other disorders (e.g., schema therapy) could have potential for negative symptoms, but there has been no research on this to date.

Depending on the individual patient the aim of γ-level interventions can be to improve physical health, reduce side effects of medication or to reduce substance abuse. Interventions targeting physical health have an increasing evidence base (see NICE guideline for current recommendations; NCCMH, 2014). In particular, physical exercise interventions have been shown to be beneficial for both physical health and negative symptoms (Firth et al., 2015). In addition to the above mentioned interventions that are more preventive in nature, it is important to treat manifest somatic illness in patients in collaboration with general practitioners and specialists (NCCMH, 2014). As outlined above, antipsychotic medication can potentially affect negative symptoms in form of sedation, bradykinesia, and amotivation. While reduction of antipsychotic dose has high face validity as an intervention to reduce these effects and is often recommended, its evidence base is limited (Kirschner et al., 2016; Aleman et al., 2017) and the question of optimal reduction strategies requires further research. Finally, there is an increasing evidence base for interventions addressing comorbid substance use in patients with psychosis (De Witte et al., 2013), which may be transferable to patients with negative symptoms.

## Interventions at the level of problematic behaviors (R)

At the level of problematic behaviors, interventions primarily involve training specific behaviors or changing the frequency of behaviors. One prominent intervention that has its origins in the treatment of depression, is Behavioral Activation (BA). Given the considerable overlap between negative symptoms and depression, it is likely that BA is also effective in reducing negative symptoms, especially problems in initiating and maintaining goal-directed behaviors. Modern BA programs are informed by individual functional analyses and include activity monitoring and scheduling, individual goal-setting as well as contingency management (see Jacobson et al., 2001; Lejuez et al., 2001) and have been proven effective compared to medication and cognitive therapy (Dimidjian et al., 2006). One pilot study examined the effects of BA on negative symptoms in a small sample of patients with schizophrenia (Mairs et al., 2011) finding some evidence for the feasibility and effectiveness of BA for reducing negative symptoms.

The most intensively studied intervention in the interpersonal skill domain is social skill training (SST) that aims at improving verbal and non-verbal communication alongside perception and responses to social cues (e.g., starting, maintaining, and terminating a conversation) (Bellack et al., 2004). SST seems to hold some promise for negative symptoms (Turner et al., 2014). However, its effectiveness seems to vary (Elis et al., 2013; Riehle et al., 2017). It thus may be dependent on additional factors. One way of improving SST could be to target the specific interactional skills that are most relevant to the individual patient after a more detailed assessment. Mostly, problems will be in the realm of affiliative and pro-social behavior (Brüne et al., 2008; Blanchard et al., 2015) rather than what is typically addressed in role-play based SST (i.e., assertiveness).

Trainings that target actual behavioral output such as smiling (a prototype affiliative signal) are rare. An example are mirror feedback techniques (e.g., Schwartz et al., 2006). A different approach could include bio- and other automated movement feedback (e.g., computerized feedback of video based measures of facial expressions) that could also target deficient movement synchronization. Moreover, body-oriented psychotherapy may improve expressive negative symptoms (Priebe et al., 2016) and improvements in interactional synchrony seem to go along with these improvements in negative symptoms (Galbusera et al., 2016).

## Interventions at the level of the consequences (C) and contingencies (K)

We argue that to optimize the learning from consequences, the whole pattern of short and long-term consequences of both the problem and the desired functional behavior along with their meaning and timing needs to be analyzed. Based on this analysis, individual schedules of reinforcement could be set up that involve reducing reinforcers of dysfunctional behavior, especially the immediate ones as far as possible and increasing immediate reinforcers of functional behavior (e.g., verbal praise, allowances, tokens that can be exchanged for individually meaningful reinforcers). For reasons of transparency and also to assure that reinforcers are meaningful for the patient, the patient needs to be involved in setting up the reinforcement plan and can also be encouraged to reinforce him- or herself. Outside of an institution, family members, who are among the most likely to be able to provide meaningful reinforcers could be involved in reinforcement schedules in combination with family interventions. Moreover, it is needs to be taken into account that short-term and immediate consequences have a stronger effect on learning new behavior, whereas intermittent consequences are more relevant to maintaining behavior. Thus, reinforcement needs to occur reliably. Well-thought contingency schedules can have an effect as stand-alone interventions but they can also boost the effectiveness of any of the interventions introduced above. The necessity of goal-setting and reinforcement of goal directed behavior has already been recognized and is included in some of the existing approaches (Medalia and Saperstein, 2011; Grant et al., 2012; Velligan et al., 2015). However, with exception of the BA approach (Mairs et al., 2011), to our knowledge, no approach so far has individualized schedules of reinforcement and personally meaningful rewards to improve on negative symptoms.

## Strengths and limitations of the FA approach for improving interventions

A main advantage of the FA approach is that it makes existing knowledge more useful to deriving therapeutic interventions and can thus help to optimize their effectiveness for the individual patient. Another strength of the approach is that it can be used flexibly. Additional factors that appear relevant to a given case (e.g., presence of comorbid disorders, a specific physical condition) can be added to the individual case-formulation and thus also be used to inform therapy.

The FA framework also opens up avenues for future research. For example, while we found abundant research on the level of the O-variable which is reflected in a higher number of intervention approaches aimed at this level, there was a dearth of research at the levels of situational triggers and consequences, which is likely to bias us toward overestimating the impact of the individual disposition and neglecting the impact of environmental factors in therapy. To inform therapy on the level of the consequences, more research is needed to understand the short-term reinforcing consequences of negative-symptomatic behavior and punishing consequences of functional and goal-directed behavior. Similarly, the framework points to gaps in assessment tools, such as the lack of standardized measures to assess reward anticipation, value representation, or effort discounting, which are required for planning individualized treatment approaches targeting reward processing.

Interestingly, there is an abundance of interventions to choose from for each of the levels. Some of these interventions outlined above are backed up by limited evidence demonstrating their efficacy for negative symptoms, while others are speculative and lack evidence. Following the logic of FA analysis, we would not necessarily expect the single interventions to show an effect on negative symptoms for a randomly selected sample of patients with negative symptoms. An important criterion for an intervention, however, is that it is successful in improving the target factor in those affected. Thus, interventions challenging dysfunctional performance beliefs should demonstrate that they can improve these beliefs in patients with these types of beliefs, affect recognition programs should improve affect recognition in those with a deficit in this domain etc. Given that each of the interventions can live up to this expectation and the clinician gets a reasonable understanding of the triggering and maintaining factors for an individual patient, a selection of the suitable interventions should theoretically lead to much stronger effects than a single intervention or a one size fits all program. To further improve treatment efficacy the clinician can attempt to take into account the feedback loops between different levels. For example, if a patient is encouraged to take more exercise to improve health-related factors at the O level this is likely to also increase the likelihood of social encounters. Using positive reinforcers at the C-level, such as praise, could not only reinforce target behavior, but also correct avoid negative self-concepts (“What does that say about your ability?”). We have provided several examples of how an individualized treatment plan based on FA may be conceptualized in the Appendix in Supplementary Material.

We are aware of the fact that the assessment and treatment planning we suggest based on the FA is time consuming. The assessments can be reduced by developing a screening-tool, which could serve as an indication for the domains for which more detailed assessments are necessary. Moreover, the additional effort for the assessment needs to be weighed against the benefits of improving outcome for severely disordered patients. Before we can do this, however, research is needed to test whether using this individualized framework will indeed produce stronger and longer lasting effects than the existing standardized interventions. This could be tested in an RCT that compares the outcomes in negative symptoms for patients who receive a standardized program to those who receive an individualized intervention based on FA.

## Author contributions

All authors discussed the approach. TL wrote the first draft of the manuscript. All authors wrote sections of the manuscript, contributed to the literature searches and provided summaries of previous research studies. All authors contributed to and have approved the final manuscript.

### Conflict of interest statement

The authors declare that the research was conducted in the absence of any commercial or financial relationships that could be construed as a potential conflict of interest.
