# Supplementary material for: Using Functional Analysis as a Framework to Guide Individualized Treatment for Negative Symptoms
Source: Front Psychol. 2017 Dec 5;8:2108. doi: 10.3389/fpsyg.2017.02108 (PMC5723417; doi:10.3389/fpsyg.2017.02108)
Supplement: Supplementary file 1 [file DataSheet1.docx]

# ***Supplementary Material***

**Using Functional Analysis as a Framework to Guide Individualized Treatment for Negative Symptoms**

Lincoln, T. M.*^[[1]](#footnote-1)^, Riehle, M.^1^, Pillny, M.^1^, Helbig-Lang, S.^1^, Fladung, A. K.^1^, Hartmann-Riemer, M.^[[2]](#footnote-2)^, Kaiser, S.^[[3]](#footnote-3)^

***Correspondence:** Prof. Dr. Tania Marie Lincoln: [tania.lincoln@uni-hamburg.de](mailto:tania.lincoln@uni-hamburg.de)

Each patient will show a unique pattern of the interplay of organism variables, responses and consequences. However, the accumulating knowledge on negative symptoms related to the situational triggers, the organism variable and the learning mechanisms, provides us with a helpful starting point on which to conceptualize cases and derive hypotheses on how negative symptoms have arisen and are maintained for any given individual. This conceptualization can then inform individual treatment planning.

## **Case example I**

### **1. Micro-level analysis**

A 20-year old patient has left hospital after a first psychotic episode and is now living at home with her family. When her older brother celebrates his birthday-party, she can be convinced to join him and some of his friends at a table in the backyard of the house. One outgoing young woman approaches her and begins a conversation (S). Based on a hypothetical FA of this situation, we could expect the patient to respond in the following way: The patient is not motivated to allocate attentive capacities to the rapidly changing non-verbal and verbal signals (O*β*) she is presented with because she has experienced difficulties with her attentional capacities (O*β*) in situations like these before. Despite reducing delusions and hallucinations her medication is making her feel drowsy (O*γ*) which adds to this problem. Furthermore, she has difficulties understanding other people’s emotions both on a perceptual and on an inferential level (O*β*). For these reasons, she has a hard time following what the other woman is saying, misses out on the emotional meaning conveyed in the messages, and is unable to respond empathically. Relevant cognitions in this situation might be “she is overwhelming me” or “I can’t keep pace with her” (R*β*). She therefore remains silent unless asked a direct question, which she replies to with short sentences (R*α*). Also, her face does not show any signs of active involvement in the conversation (she does not smile and does not nod appropriately) (R*α*). At first, this behavior motivates the other woman to try harder, non-verbally, making herself clear (reinforcer and new S). In addition to the O-variables outlined above, now her difficulties in processing social reward signals (the encouraging smiles of her interaction partner) prevent her from experiencing the other woman’s behavior as rewarding. Her defeatist performance beliefs (O*β*) become activated and result in her being ashamed and in cognitions such as “I don’t want to respond inadequately” or “I’m a social mess” (R*β*). Behaviorally, she is now also constantly avoiding eye-contact (R*α*). The outgoing woman finally gives up and turns to another person at the table. The patient feels relieved (negative reinforcer). Her interaction partner will, however, not try to approach her again (long-term punishment by contingent withdrawal), which is likely to increase loneliness. On the next day, her brother criticizes her for not having made more of an effort to chat with the woman (punisher), which makes her feel even more uncertain in regard to her interactional skills (long-term punisher and feedback to O*β).*

### **2. Potential interventions derived from the FA for this case**

We now describe a possible line of treatment for this patient presuming that the difficulties described in the vignette were reliably assessed. The primary treatment target for this patient would be to reduce the cognitive load of social situations (O*β*), since the patient perceives them as overwhelming and for this reason reduces her engagement. Secondary treatment targets would be to reduce defeatist performance beliefs (O*β*) that become activated in form of dysfunctional automatic thoughts as a result of the patient’s experience with her difficulties in social situations (R*β*) and to reduce the criticizing behavior of the immediate social support network (here the family) (confirming her defeatist performance beliefs, her withdrawal behavior, and her motivational deficit).

A first line of treatment could exist in reducing the sedative effects of medication (drowsiness) that contributes to the overwhelming character of social situations. This could involve switching of antipsychotic or dose reduction. Re-emerging positive symptoms could be dealt with using the evidence-based psychological aimed at reducing delusions and hallucinations, specifically (Van der Gaag, Valmaggia, & Smit, 2014). In the psycho-social realm, for this patient, components 2 and 3 from the MOVE program proposed by Velligan and colleagues (2015) that address the social cognitive and the expressive deficit could help the patient to overcome the difficulties in processing social input effectively. Accompanying cognitive interventions could be used to address the defeatist performance beliefs and the motivational problems. Particularly behavioral experiments would be helpful for the patient to understand the differential consequences of behaving in one or another way (e.g. keeping or not keeping eye contact) and to test her anticipation of pleasure in the activities. Since this patient has sufficient cognitive capacity, disputing her defeatist beliefs and automatic thoughts is a practical choice. In addition to these individually oriented therapeutic techniques, a group based social skills training for schizophrenia would provide the patient with additional training in social situations. A collaborative psycho-educational approach that uses the FA model and involves both the patient and her family members may help both the patient and her immediate social environment to better understand the specific interactional problems and reduce self-blaming on sides of the patient and criticizing behavior on sides of the family. Additionally, it may prime the family members to pay extra attention to the changes that result from the therapy and to reinforce respective behavior with reinforcers that are known to work for the patient.

## **Case example II**

### **1. Micro-level analysis**

A patient with severe symptomatology in both the motivational and the expression domain is sitting on his own in the corner of the common room of an outpatient day-care facility, when he is approached by a nurse asking him to get up and come and join the activity group (S). The patient responds to this request by muttering “no thanks”, remains seated and shows no mimics or physical signs of interest or regret of not attending (R*α*). The patient’s difficulties in anticipating feelings of pleasure in expectation of the social activity (O*β*), result in automatic thoughts such as “It won’t be fun” and “It’s not worth the bother” (R*β*). Due to physical conditions related to cardiovascular health problems and obesity (O*γ*), he also thinks “I don’t have the energy” (R*β*). Finally, due to negative self-concepts and a low expectation of self-efficacy (O*β*) he further assumes that he will not have anything to contribute and will make a fool of himself (R*β*). This assumption goes along with the corresponding psychophysiological indicators of arousal (R*γ*), which he perceives as a brief feeling of sadness or even anxiety.

The patient’s response has a number of short and longer-term consequences: For one, the decision not to go produces a short-term sense of relief (i.e. reduction in arousal) because of not having to expose himself to an activity that goes along with effort and bears the risk of rejection and shame (*negative reinforcer*). Another short-term consequence is that the nurse shows signs of disapproval (“You should be making more of an effort”), and even expresses rejection by commenting “Don’t be lazy” (*punisher*). On a longer term, the nurse gives up on the patient and stops asking him to join (*negative punisher*). Another direct consequence is that the patient misses out on an opportunity to connect with others (*negative punisher*) that would have corrected some of his dysfunctional expectations about social encounters and made him feel better about himself (feedback mechanisms).

### **2. Potential interventions derived from the FA for this case**

For this patient, the treatment targets would be to increase participation in common daily activities in the clinic in order to address the resulting consequences of inactivity, for instance criticism and poor physical health. Interventions on the S-, O-, and C-variables may be particularly beneficial for this patient, as his inactivity appears to be influenced by problems in anticipation and expression, defeatist performance beliefs, and social anxiety. Accordingly, the burden to put effort in an activity is even higher when it is a group activity. Thus, on the level of the R-variable, the therapist and the patient would look for activities that he can pursue on his own at first and ones that require little effort and re reinforcing. The effort burden could then be increased over time. If the patient can be motivated to engage in such activities alone and feels comfortable with this, he might be convinced to join group activities later on.

To foster participation in group activities the defeatist performance beliefs (O*β*) can be addressed with CBT techniques (e.g. Beck et al., 2013). Again, this should reduce the effort that the patient feels is required to engage in a group activity (O*β*). Similar techniques could be used to address convictions that participation is not worth the effort (O*β*). These could be supplemented with behavioral experiments and diary techniques that could help the patient to make more positive predictions about participating in daily activities.

On the level of the C-variable, the staff could be encouraged to avoid open displays of disappointment and criticism when confronted with reluctance. Additionally, the staff could be sensitized to not reduce interpersonal efforts in response to the patients not responding to their offers. Instead, small steps in direction of goal-directed behavior should be rewarded within an individual contingency management plan (shaping). These interventions may also make the patient’s living environment more stimulating (S-variable).

## **Case example III**

### **1. Micro-level analysis**

A 41-year-old patient with a 20-year history of schizophrenia, who takes antipsychotic medication regularly and lives on a disability pension has slept in, made himself a cup of coffee and is sitting at the kitchen table to decide what he wants to do during the day. He realizes that a social event at the soccer fan club will take place for which he is supposed to bring a salad (S). Due to concentration and attention deficits, he has a hard time planning the steps necessary to be able to prepare the salad (O*β*). Moreover, he cannot come up with any concrete ideas for a salad (option generation; O*β*). His problems in reward anticipation (O*β*) make it hard for him to picture the pleasure of accomplishment of having prepared a salad. Moreover, he is not quite sure he is even able to prepare it (low self-efficacy; O*β*). He begins to feel tense (R*γ),* accompanied by thoughts such as “I won’t be able to manage this” (R*β*). In order to calm down, he smokes some marijuana (R*α*), and feels an almost immediate relief (negative reinforcement). The smoking (new S) and the corresponding drug effects (O*γ)*, however, increase his problems in attention and goal-setting (O*β)*, causing him to ruminate about whether it’s worthwhile going at all “It’s not worth the effort; I won’t make it anyway” (R*β*). Thus, he decides to cancel the appointment and turns on the TV (R*α*). He is distracted from his negative thoughts (negative reinforcement), and avoids a potentially frustrating experience (negative reinforcement). In the long-run, his fan mates are disappointed because he did not show up and may not invite him again (long-term punishment by contingent withdrawal). Furthermore, the patient’s defeatist performance beliefs are strengthened (long-term punishment) and he is more likely to refuse participation in social activities, leading to social withdrawal.

### **2. Potential interventions derived from the FA for this case**

The micro-analysis for this patient provides several treatment targets: First, at the level of R, the consumption of marijuana should be reduced if possible as it aggravates the patient’s neurocognitive and volitional deficits. Second, at the level of O, interventions should aim at improving the patient’s overall perception of self-efficacy as well as cognitive abilities.

The first aim might be targeted by interventions such discussing the implicatins of marijuana on the basis of the FA and by using motivational interviewing to increase the patient’s willingness to refrain from drug consumption, and behavioral techniques such as relaxation training to provide the patient with an alternative for calming down. Moreover, interventions aimed at improving attention, concentration, planning and decision making should be selected. For basic functioning, cognitive remediation (e.g., Cognitive Enhancement Training (Eak, Mesholam-Gateley, Greenwald, Hogarty, & Keshavan, 2013) might be used. The patient might further benefit from a formal problem-solving training and a self-instruction training in order to increase the ability to generate intentions and put them into practice in a given time-frame. This could be combined with a behavioral activation program that increases the rate of potentially rewarding activities and provides him with the opportunity to set goals and put them into practice, which should foster the patient’s sense of control and self-efficacy.

Eak, S. M., Mesholam-Gateley, R. I., Greenwald, D. P., Hogarty, S. S., & Keshavan, M. S. (2013). Negative symptom improvement during cognitive rehabilitation. Results from a two-year trial of cognitive enhancement therapy. *Psychiatry Research*, *209*(1), 21–26. https://doi.org/10.1016/j.psychres.2013.03.020

Van der Gaag, M., Valmaggia, L., & Smit, F. (2014). The effects of individually tailored formulation-based cognitive behavioural therapy in auditory hallucinations and delusions: A meta-analysis. *Schizophrenia Research*, *156*, 30–37.

Velligan, D., Roberts, D., Mintz, J., Maples, N., Li, X., Medellin, E., & Brown, M. (2015). A randomized pilot study of MOtiVation and Enhancement (MOVE) training for negative symptoms in schizophrenia. *Schizophrenia Research*, *165*(2–3), 175–180. https://doi.org/10.1016/j.schres.2015.04.008.

1. Clinical Psychology and Psychotherapy, Institute of Psychology, Faculty of Psychology and Movement Sciences, University of Hamburg, Hamburg, Germany [↑](#footnote-ref-1)
2. Department of Psychiatry, Psychotherapy and Psychosomatics, Psychiatric Hospital, University of Zurich, Zurich, Switzerland [↑](#footnote-ref-2)
3. Adult Psychiatry, Geneva University Hospitals, Geneva, Switzerland [↑](#footnote-ref-3)
